# Emotion, Morality, and Interpersonal Relations as Critical Components of Children’s Cultural Learning in Conjunction With Middle-Class Family Life in the United States

**DOI:** 10.3389/fpsyg.2019.01456

**Published:** 2019-06-26

**Authors:** Karen Gainer Sirota

**Affiliations:** Department of Human Development, California State University, Long Beach, Long Beach, CA, United States

**Keywords:** children, culture, emotion, family, language, morality, socialization

## Abstract

An enduring question in the cultural study of psychological experience concerns how emotion may play a role in shaping moral aspects of children’s lives as they are mentored into socially preferred ways of understanding and responding to the world at hand. This article brings together approaches from psychological and linguistic anthropology to explore how cultural schemas of normativity are communicated, embodied, and enacted as children participate in day-to-day family activities and routines. Illustrative examples emanate from a videotaped corpus of naturalistic interactional data that document the daily lives of 32 ethnically diverse U.S. middle-class families who reside in the Los Angeles, California metropolitan region. The article employs discourse and narrative analysis to examine how children are apprenticed into perceiving, appraising, and reacting to the emotions of self and others as culturally shaped indicators for proper comportment. Data analysis emphasizes how implicit components of caregivers’ interactions with children (i.e., gesture, gaze, facial expression) intertwine with explicit, verbal communication to constitute intricately layered affective messages that shape the evaluative frames through which children interpret, display, and respond to emotions. The article identifies two culturally salient childrearing practices, “pep talks” and “time outs,” that apprentice children into moral accountable relationships with others by encouraging them to manage their emotions in culturally preferred ways. Study findings suggest that parental communications conveying praise and approval—or conversely indexing disapproval—toward children are emotionally resonant motivational practices in this cultural milieu as children are mentored into culturally meaningful emotional management techniques. The article highlights how children actively employ semiotic socio-communicative resources and it closely traces their sense-making processes in tandem with their discursive contributions to the moment-by-moment interaction. It argues that emotion, morality, and interpersonal relations are critical in shaping children’s acquisition of consensually validated ways of perceiving, feeling, and responding to the phenomena they encounter in their day-to-day lives. This perspective aims toward contextualized understandings that render plausible connections between local contexts of everyday action and broader macro-level discourses and master narratives, such as those associated with a neo-liberal emphasis on cultivating citizens who learn to regulate their emotions on behalf of self and others.

## Introduction

An enduring question in the cultural study of psychological experience concerns how emotion may play a role in shaping moral aspects of children’s lives as they are mentored into socially preferred ways of understanding and responding to the world. The current study brings together approaches from psychological and linguistic anthropology to explore how cultural schemas of normativity are communicated, embodied, and enacted as children participate in day-to-day family activities and routines. The article examines how U.S. middle-class children are apprenticed into perceiving, appraising, and reacting to the emotions of self and others as culturally shaped indicators for proper comportment. Data analysis identifies two culturally salient childrearing practices, “pep talks” and “time outs,” that apprentice children into moral accountable relationships with others by encouraging them to manage their emotions in culturally and socially preferred ways. Study findings indicate that parental communications that convey approval and praise—or, conversely, index disapproval—toward children are emotionally resonant motivational practices in this U.S. middle-class cultural milieu. The article explores how parents employ emotions to convey and model culturally salient moral values to children. It also addresses how these childrearing practices socialize children into culturally pertinent moral norms and techniques of emotion expression and regulation. It further proposes that these two components of socialization go hand-in-hand and occur in tandem with one another. The emotional meaning and salience of the parent-child relationship thus shape the motivational and contextual frame in which this socialization unfolds.

### Morality and Emotion in Everyday Life

Endeavors to arrive at contextualized understandings about how morality is shaped and enacted amidst everyday life circumstances, *in situ*, have come to the fore in recent years as counterpoints to the relatively abstract, circumscribed approaches that have guided, in large measure, contemporary social scientific outlooks about ethics and morality, such as those that draw on fixed developmental sequences (e.g., Piaget, 1932; Kohlberg, 1981); universal psychological and/or social principles (e.g., Gilligan, 1982; Turiel, 1983); structurally determined normative ideals (e.g., Parsons, 1951; Habermas, 1990); institutionally or culturally endorsed rituals and customs (e.g., Durkheim, 1965; Goffman, 1967); or philosophically derived, ubiquitous ethical “goods” (e.g., Rawls, 2001; MacIntyre, 2007).

In contrast, recent explorations in the arenas of linguistic, medical, and psychological anthropology, cultural and discursive psychology, and conversation analysis point to the utility of approaching the study of morality as it is situated and negotiated amid the vicissitudes of everyday practice (e.g., Shweder and Much, 1991; Bergmann, 1998; Briggs, 1998; Kleinman, 1998, 2003; Shweder, 2003, 2012; Sterponi, 2003, 2009; Fung, 2006; Goodwin, 2006; Kleinman, 2006; Zigon, 2007, 2014; Parish, 2008, 2014; Ochs and Izquierdo, 2009; Lambek, 2010; Sirota, 2010a; Throop, 2010, 2014; Heritage, 2011; Stivers et al., 2011; Miller et al., 2012; Demuth, 2013; Fassin, 2013; Mattingly, 2013, 2014; Ochs and Kremer-Sadlik, 2013b; Takada, 2013; Desjarlais, 2014; Garcia, 2014; Willen, 2014; Goodwin and Cekaite, 2018). Such fine-grained, culturally attuned analyses provide nuanced, on-the-ground portrayals of people’s pragmatic engagements in “local moral worlds” (Kleinman and Kleinman, 1991, p. 277) in which people stand to differentially gain or lose in matters that involve closely held values, pursuits, relationships, ideas, material conditions, and the like. This research sheds light on the reciprocal co-constitutive processes via which individuals’ moral choices, experiences, and outlooks are cultivated and shaped in dynamic interaction with sociocultural norms. In stressing the importance of deriving contextualized and ecologically valid understandings about morality, cultural psychologist and anthropologist Shweder (2012) argues for the importance of cross-cultural descriptive fieldwork that explores the local nuances of moral precepts, judgments, and views. Moral endeavors are lodged, and transpire, within specific interactional contexts, temporal trajectories, cultural settings, and socio-political circumstances (Goodwin, 2006). Individuals’ responsibilities, virtues, rights, transgressions, accomplishments, and trajectories of conflict or cooperation are thus calibrated and attuned in dialogue with others. It is therefore crucial to attend to how morality is enacted and takes shape in real world surroundings and situations. As such, “morality is not transcendent, but always embedded in the need to sustain relations with others” (Lutz, 1988, p. 77).

The perspectives outlined above inform the approach I adopt in this article to the study of moral discourse and moral action. I also take inspiration from insights provided by anthropologist Zigon (2014) regarding the salience of contextualized, process-oriented inquiry into human moralities as they are lived and transacted amid the contingencies, ambiguities, and uncertainties of day-to-day life. Zigon proposes an ontological standpoint, adapted from Heidegger (1996), which construes “being and the world as coeval” (Zigon, 2014, p. 20; see also Evens, 2005). On this view, human actors are “always already entangled in a multiplicity … of relationships that deeply matter for their very existence as subjects” (Zigon, 2014, pp. 21–22). These existential conditions, suggests Zigon, set the stage for an expansive web of meaningful engagements in which the ethical projects of self and others hold pertinence for one another as each party actively engages past, present, and future possibilities, prohibitions, imaginaries, and constraints. “Morally being-in-the-world,” Zigon further notes, entails “nonconscious embodied modes of moral life” as well as moments of conscious ethical reflection that hold transformative potential (Zigon, 2014, pp. 24, 18).

These morally laden communicative processes are simultaneously self-shaping and relational. Parish (2014), for example, draws attention to the intersubjective contours of morality. Moral transactions are thus negotiated in the “space between persons” (Parish, 2014, p. 33). Moreover, they are coupled with the existential challenges of humanely responding to other persons, and of meaningfully grappling with the presence of others in ways that have “experiential and emotional force” (Parish, 2014, p. 37). In their cross-cultural language socialization study of moral responsibility among Samoan, Peruvian Matsigenka, and middle-class Los Angeles, California, families, anthropologists Ochs and Izquierdo (2009, p. 391) stress the import of socializing and supporting children’s proclivities for “active(ly) turning toward the other” so as to foster their “awareness of and responsiveness to others’ needs and desires.” Morally accountable actions, choices, and calibrations yield consequential relational effects that involve affiliation, cooperation, and alignment with others, as well as disagreement, resistance, and power asymmetries vis-à-vis social relationships (Stivers et al., 2011).

Moral encounters in daily life transpire within communicative contexts that call upon co-participants to intelligibly utilize, recognize, and interpret multimodal linguistic and paralinguistic cues so as to orchestrate mutually understood, culturally valued ways of being, believing, and behaving. Trevarthen (2011, p. 123) emphasizes the crucial role of emotion in establishing and negotiating intersubjectively shared meanings, principles, and values. It is “human feelings,” Trevarthen observes, “that give *aesthetic appraisal* of things and events, and *moral appraisal* of others’ actions.” From their earliest moments as newborns, children are provided with a richly elaborated set of cultural resources that interpretively frame their subjectively experienced emotions as well as the emotions they observe in others (Demuth, 2013, p. 41). As such, children are mentored into culturally pertinent techniques of heeding and deriving meaning from the emotions of self and others. Culturally elaborated emotional experiences, expressions, and responses index and convey moral stances about what is culturally valued or denigrated, normative or deviant, decorous or improper, soothing or abrasive, and so on (Sirota, 2018). Culturally mediated “socializing emotions” (Röttger-Rössler et al., 2016) conveyed in conjunction with emotionally charged childrearing practices—such as shaming, praising, teasing, frustrating, admiring, adoring, frightening, and disapproving—communicate and reinforce memorable lessons to children about preferred values, norms, dispositions, and tastes (e.g., Briggs, 1970, 1998; Miller, 1982; Miller and Sperry, 1987; Fung, 1999; Quinn, 2005; Willhinganz and Wingard, 2005; Sirota, 2010a; Miller et al., 2012; Röttger-Rössler et al., 2016). Goodwin and Cekaite (2018, p. 122) call attention to the multimodal, embodied components of such socioemotional communications between children and caregivers, which are constituted through an “interactive sensorium” that comprises various components of speech, such as vocal content, quality, and prosody, yet also encompasses haptic, corporeal characteristics such as gesture, gaze, touch, and bodily positioning. These contemporary insights about the pertinence of interactional micro-processes in transmitting culturally valued ways of understanding and responding to the world-at-hand are prefigured and supported by the pioneering work of Margaret Mead and her collaborators (e.g., Bateson and Mead, 1942; Mead and Macgregor, 1951; also see Bateson, 1972), whose closely detailed ethnographic observations and photographic analyses highlight the interactional relations among affective, communicative, sensorimotoric, and interpersonal aspects of children’s cultural learning.

The approach adopted in this article regards cultural learning as a situated activity that involves participation in locally unfolding sequences of interaction that transpire in conjunction with socioculturally informed “communities of practice,” such as nuclear and extended family constellations, peer groups, schools, and religious congregations, among others (Lave and Wenger, 1991; Erickson, 2002). Psychologist and educator (Rogoff, 2003; Rogoff et al., 2018) emphasizes that “learning and development occur in the process of people’s participation in the activities and events of their cultural communities” (Rogoff et al., 2018, p. 6). However, it is crucial to note that cultural communities are not uniformly homogenous; rather they entail intracultural variation among community members (Strauss and Quinn, 1997; Strauss, 2012; D’Andrade, 2018; Quinn et al., 2018). Moreover, an individual may be part of multiple communities of practice, concurrently and/or successively across various points in the life course. Thus the development of particularized cultural competencies, values, identities, and worldviews is contingent upon which distinctive communities of practice an individual encounters as well as on the particularized fashion that each community of practice encourages “apprentice-like learning of certain patterns of conducting everyday life” (Erickson, 2002, p. 302).

Significantly, as well, various cultural communities differ in how they formulate and ascribe meaning, purpose, and value to biologically rooted emotional states and inclinations. The current study follows cultural psychologist Miller and colleagues (e.g., Miller and Sperry, 1987; Miller et al., 2007, 2012) in positing that emotion is neither solely nor unequivocally biological in nature; emotion is also mediated by—and responsive to—sociocultural norms. For example, emotions may be hypocognized or hypercognized in conjunction with culturally conditioned patterns of attentional focus that involve perception, attribution, lexicalization, and/or interpretation of feeling states (Levy, 1973, 1984). Also of note are culturally informed “feeling rules” (Hochschild, 1979, 2012) that tacitly and explicitly guide “who may feel which emotions when, with which intensity, and in front of whom they should be expressed or suppressed” (Röttger-Rössler et al., 2016, p. 187). “Children’s developing expression of emotions,” Miller and Sperry (1987, p. 2) likewise propose, “is influenced by the culturally patterned assumptions about emotional life that parents intentionally and unintentionally communicate to them.” For example, cultural psychologist Fung et al. (2004) call attention to the contrasting cultural styles of emotion and morality into which young European-American and Taiwanese children are socialized through personal storytelling with their parents. Moral transgressions of Taiwanese children are highlighted and made emotionally salient through personal stories that inculcate feelings of shame about said transgressions. By comparison, European-American middle-class children are narratively positioned so as to valorize children’s feelings of self-affirmation and self-esteem. In a related vein, psychocultural anthropologist Briggs (2000, p. 160) emphasizes that, “the repertoire of emotions is not the same the world over.” Rather, emotions and their meanings are always embedded within, and contingent upon, the contexts in which they arise and take shape. Emotional meanings and purposes, Briggs (2000), pp. 160–161) suggests, are lodged within associative webs that draw upon and encompass community members’ past, present, and future values, priorities, and conceptions about human nature and the bounds of proper comportment.

Briggs (1970), Briggs (1998, p. 207); Briggs (2000), Briggs (2008) ethnographic research among Inuit children and families examines how children learn to puzzle out, engage, and reconfigure “labyrinths of meaning” in which emotion and morality are at stake, and in which socially preferred attitudes, emotions, and behaviors intertwine in culturally coherent ways. Briggs (1998, p. 203) research attunes us to important questions about the type of work emotions do in day-to-day social life. One such query is posed by Briggs (1998, p. 204): What kinds of experiences foster, support, or transform cultural “patterns of thinking, feeling, and valuing?” Furthermore, how—and what—do children learn about culturally configured emotions that potentially ease social relations or, alternatively, draw people apart? I take up these questions in the remainder of this article through an examination of two culturally recognized U.S. middle-class childrearing practices that are evident in the CELF project’s research data: “pep talks” and “time outs,” as each are colloquially termed. Both practices are aimed toward cultivating children’s ability to restore their emotional equilibrium following a perturbation due to untoward adversity or distress. The data analysis that follows illustrates how these practices of U.S. middle-class family life play a role in mentoring children’s accountability to others by encouraging them to manage their emotions, as well as actions that flow from these emotions, in culturally and socially preferred ways.

## Materials and Methods

### Research Aim, Recruitment, and Participants

The research data analyzed in this study are part of a larger data set collected between the years of 2002 and 2005 by the University of California, Los Angeles (UCLA) Sloan Center on Everyday Lives of Families (CELF). The CELF research project aims to shed light on the socio-communicative processes and interactional structuring of U.S. middle-class dual earner families by closely documenting the daily activities and routines of 32 ethnically diverse, middle-class dual earner families residing in the metropolitan area of Los Angeles, California.

Participating families were recruited via informational fliers that were distributed in local schools as well as through advertisements in community newspapers. Limited snowball sampling was also employed. Several families (*n* = 4) were thus recruited to the study by word of mouth after learning about the research from CELF project participants who were their neighbors or friends. Research parameters necessitated that families met all of the following criteria for inclusion in the study: (1) two parenting adults, each of whom worked at least 30 h weekly outside the home; (2) two or three children, at least one of whom was between 7- and 12-years of age; (3) self-identified middle-class status that included owning (or holding a mortgage on) a single-family home. Each family received financial compensation ($1,000 U.S.) for their study participation.

The ethnic diversity of participating CELF families approximated, and roughly mirrored, the diverse demographic composition of the greater Los Angeles metropolitan region. Parenting adults who participated in the CELF research study self-identified as follows: Hispanic (6%); African American (8%); Asian and South Asian (14%); Caucasian Non-Hispanic (72%). CELF mothers ranged between 32 and 50 years of age (mean = 40 years). By comparison, CELF fathers’ ages ranged from 32 to 58 years (mean = 42 years). The vast majority (67%) of participating parents were college educated. CELF parents’ occupations were quite diverse (i.e., teacher, office clerk, technician, lawyer, fireman, business owner, social worker, accountant). However, CELF families’ household income median ($115,000 U.S.) exceeded the contemporaneous income average of families residing in Los Angeles (cf. Ochs and Kremer-Sadlik, 2013a) and likely reflected CELF parents’ relatively advanced educational attainment. CELF children’s ages spanned from 1- to 18-years old. As was noted above, the family composition of each participating family included at least one “target” aged child who was between the ages of seven and twelve, so as to ensure that all CELF families were in a comparable stage of their “family life cycle” (McGoldrick and Carter, 2005) in which childrearing plays a key role. (For additional demographic details about the CELF study population, see Ochs and Kremer-Sadlik, 2013a).

### Ethics Statement

This research was conducted in accordance with the recommendations of the Institutional Review Board at the University of California, Los Angeles (UCLA). The research protocol was reviewed and approved by the UCLA Institutional Review Board (UCLA IRB Protocol #G01–06–083–14). In accordance with the World Medical Association’s Declaration of Helsinki, written informed consent was provided by, or on behalf of, all research participants. Written informed consent was obtained from all adults who participated in the research and from the parents of all non-adult research participants under the age of eighteen. Written informed assent was obtained from all non-adult research participants between the ages of seven and seventeen. Study participants’ names have been disguised to safeguard confidentiality as per IRB-approved research protocol.

### Data Collection, Transcription, and Analysis Procedures

The CELF study’s multi-modal compendium of research methods closely tracked and documented a proverbial “week” in the life of each working family who participated in the research project. Data collection methods included naturalistic observations of family members’ household activities and routines, semi-structured interviews and self-report measures provided by parents and children, and physiologic stress measures of family members’ salivary cortisol levels. The research data employed in this article are drawn from the study’s archive of observational and interview data.

Center on Everyday Lives of Families researchers conducted observational videotaping with each family over the course of a week, using digital video cameras outfitted with wide-angle lenses and remote microphones that allowed researchers to position themselves discreetly at a distance from research participants. Two researchers simultaneously videotaped family members as they carried out their daily activities, while a third researcher tracked and notated each person’s activity involvement and use of space within the home at 10-min intervals. Observational data collection transpired on four separate days (2 weekdays, plus Saturday and Sunday), and commenced in the morning when family members awoke. On of these 3 days (2 weekdays, plus Sunday), the researchers also returned to document family members’ afternoon and evening activities up until the time the children went to sleep. Approximately 50-h of videotaped observational data were collected for each participating CELF family. In addition, CELF researchers conducted video- and audio-taped interviews with family members on a range of topics including daily routines, social networks, education, health, and attitudes toward work and family.

Family members were introduced to the video cameras prior to the time that formal data collection procedures began. This helped them become accustomed to the presence of researchers with digital recording equipment in their home. CELF participants’ initial reactivity and self-consciousness about being videotaped diminished as they acclimated to the CELF research procedures. In addition, the prolonged duration of the video data collection in each family decreased any inclination for participants to sustain an ongoing “performance” that was purposively geared toward displaying socially desirable behavior.

The study’s ethnographic, observational research methods are designed to document the naturalistic ebb and flow of family life as it spontaneously occurs, moment by moment. CELF data collection procedures sequentially chronicle what participants do and say, how and where they position themselves in relationship to their material and ecological surroundings, and how they orient and respond to each other.

All video- and audio-taped data were digitized, logged, and transcribed by CELF research assistants using vPrism computer software that allows for synchronization of the video images and audio tracks with the accompanying written record of the discourse phenomena. Data were transcribed employing conversation analytic transcription conventions (adapted from Atkinson and Heritage, 1984). The author (KS) subsequently completed an additional, more fully detailed transcription of the discourse data analyzed and discussed in this article to facilitate the identification of relevant discourse phenomena and to enhance the granularity of the data analysis (see Appendix).

The fine-grained transcription and analysis of discourse data illuminates pertinent, sequentially unfolding features of talk-in-interaction. These include people’s spoken words, syntax, vocal timbre, pitch, and prosody, facial expressions, eye gaze, and embodied posture and positioning as they interface with pertinent features of the material and ecological surround (Goodwin, 2013, 2018).

Additional rounds of data coding also were carried out by KS using an inductive “grounded theory” data analysis approach (Corbin and Strauss, 2008; Glaser and Strauss, 2017) to identify pertinent interactional features and patterns that empirically and organically emerged from the data. This approach accords with a discourse analytic perspective in that both methodological frames foreground research participants’ orientations, actions, and understandings with respect to the interactions in which they are engaged. The families and discourse excerpts selected for discussion and analysis in this article constitute representative exemplars that illustrate broader patterns in the larger research data corpus.

The discourse analytic perspective I employ in this article considers talk-in-interaction as a vital compendium of socio-communicative practices that are instrumental in formulating and shaping historically and socioculturally rooted dispositions, values, stances, and tastes—and that structure plausible fields of action in conjunction with political, economic, and social potentialities and constraints (cf. Goodwin, 2000, 2013; see also Sirota, 2010b, 2018). This approach documents and analyzes salient aspects of interactional process and content. The article also explores children’s active roles, contributions, and participation in cultural learning through analytic vantage point of language socialization (cf. Ochs and Schieffelin, 1984, 2011), which attends to the co-constructed communicative processes involved in apprenticing children’s cultural competencies, values, identities, dispositions, and worldviews—and in which children and mentors each actively contribute.

#### Analysis and Discussion

The data findings and interactive data sequences detailed in this section exemplify two distinctive socialization practices for morally accountable emotion management that are evident in the CELF data corpus and that CELF research participants, from their own perspective, emically term, “pep talks” and “time outs.” It bears noting that both of these terms (“pep talk” and “time out”) are also commonly used in the American sporting domain. (This athletic parlance is a possible source for this terminology’s appropriation into the U.S. middle-class family sphere). It also is important to note that a number of highly effective childrearing practices in the United States and elsewhere are less explicitly cognized and lexicalized as compared with the pep talk and time out practices that are identified and discussed here (cf. Quinn et al., 2018; Sirota, 2018). The data examples and families selected to illustrate these discursive phenomena in the section below are representative of broader findings evident in the CELF data corpus as a whole.

A total of 213 “pep talk” and “time out” sequences were identified across the entirety of the CELF video data corpus. Included in this total are a sub-corpus of 106 “pep talk” sequences and a sub-corpus of 107 “time out” sequences. “Pep talks” and “time outs” were fairly evenly distributed across the research sample of 32 participating families. “Pep talks” and “time outs” both were evident in 84.38 percent of CELF families (*n* = 27). Three CELF families (9.37%) employed “time outs” but did not make use of “pep talks” during the study observation period. By comparison, two CELF families (6.25%) used “pep talks” but did not employ “time outs.” Notably, however, all 32 CELF families employed at least one (if not both) of these practices during the data collection period. The number of “pep talk” sequences per family ranged between zero and twelve (mean = 3.31) whereas the number of “time out” sequences per family ranged between zero and ten (mean = 3.34). 75 children (ages one to sixteen) participated in the CELF study. The mean number of “pep talks” per participating child was 1.41. In comparison, the mean number of “time outs” per participating child was 1.43. Albeit, the use of “pep talk” and “time out” sequences was more prevalent among CELF school-aged children and young adolescents (ages five to thirteen) in comparison with younger CELF toddlers and pre-school-aged children (ages one to four) and CELF adolescents (ages fourteen to sixteen). As will be further explored in the data analysis and discussion sections that follow, these latter findings suggest that CELF parents held differing developmental expectations regarding emotion management and the use of emotion management socialization practices with relatively younger versus older children.

### Data Examples: “Pep Talks” and “Time Outs”

“Pep talks” are intended to soothe children’s ruffled emotions during a moment of distress while simultaneously boosting children’s feelings of efficacy and self-confidence. For purposes of this study, “pep talks” are differentiated from more generalized parental compliments to children in that pep talks involve extended interactional sequences that span two turns at talk or more by parent and child, respectively. Moreover, pep talks commenced in response to a child’s verbally articulated (and/or corporeally displayed) disappointed, dejected, or saddened mood. During pep talk sequences, parents validated—and commiserated with—children’s upset feelings. In addition, parental reassurance aimed toward instilling a sense of hope so as to encourage children to move forward and take further action toward resolving the problem at hand. As one CELF father, Adam Lear (with two daughters, ages eight and six), explained during a research interview:

It’s important for kids to learn how to not get too upset over small things. I tell my daughters: “It’s a waste of your energy to be ***crying*** over something like this. Try to turn that energy into something you need to do for the next half an hour. Not crying.” It’s the same idea as with lemons and lemonade, you know? You just want to ***change*** the mood for them. I push them to do something that will help them feel ***good*** about themselves so they are moving in the direction of staying happy, and positive, and whatever. By ***applying*** their ability to make decisions and choices and change. Which then translates into feeling good, and healthy, and well ***balanced***. All that kind of stuff. Not to go the other way and be stuck and angry and frustrated, you know?

Likewise, in another CELF family, when 7-year-old Michael Reis pouts about not being chosen to play the “goalie” position in his upcoming ice hockey match and consequently threatens to quit the game, his parents Jerry and Pam initiate a pep talk to calm and reassure him. “Do I ***have*** to go? I like being goalie ***more***!” Michael implores in a frustrated voice tone. “I know you do,” acknowledges Jerry. “But this will be an ***opportunity***. Just try it for a day. It’ll be fun,” he optimistically suggests. “Why don’t you want to go?” Pam inquires of her son. “I can goalie ***better***!” Michael insistently counters. Pam then provides additional encouragement and proffers a suggestion to reframe the situation: “You’re going to be an amazing skater today because you’re not going to have all that extra weight (protective goalie gear) on you. You can skate out for one day. You can be the ***assistant*** goalie. And help out the goalie.” Michael enthusiastically endorses this idea. His countenance brightens as he fetches his hockey stick and imaginatively enacts assisting a teammate block a goal. Michael’s mood is thus buoyed as Pam and Jerry provide offer him words of encouragement and urge him to pursue options that will help him to successfully participate in the ice hockey game.

In a CELF household nearby, 8-year-old Jack Walters is similarly encouraged and spurred on by a parent-child “pep talk” after he expresses feelings of disappointment about the grades he earned on his report card. Jack ruefully hangs his head and heaves a heavy sigh, as he wistfully informs his parents, “I thought I was going to do better. I thought I was going to at least get some fours on my report card.” Jack’s mother, Lila, offers immediate words of comfort to her son. “You’re ***so*** smart. It’s not that you’re not ***capable***,” she reassuringly suggests. “We’d like to see you apply yourself to your ***potential*** so you can get the grades you’re capable of” Lila adds. Jack’s stepfather, Matt, chimes in to provide added support. “We’re so proud of you,” he proclaims. Jack’s palpable relief is evident in his physical demeanor as he sits upright and smiles broadly in response to his parents’ morale-boosting comments.

In contrast to “pep talks,” during which family members huddle around a distraught child, “time outs” entail an imposed period of solitary quiescence during which a child is temporarily separated from the ongoing stream of social activity in order to interrupt and calm an infelicitous outburst of untoward behavior and emotion. In such instances, parents use the emotional meaning and salience of their relationship with children as leverage toward attaining children’s compliance with culturally identified norms of emotion expression and regulation. As CELF father-of-two, Kent Yokoyama, recounts during an informal interview with researchers:

Sometimes the kids get moody. They have their- sort of emotional ups and downs. And they’ll get a little whiney, you know? And ***that*** doesn’t help when they have to interact. First, stop your whining. Go take some time out and calm down. And ***then*** we’ll listen. And the world’s not ending. So just keep breathing and you’ll survive, you know? Like the other day when Kei (8-year-old daughter) was whining and fussing. I said to her, “Shush Kei, go inside! It’s time to stop ***whining***. Go inside and ***settle down***.”

An additional illustration of such “time out” principles arises in another CELF family when 9-year-old Hannah Friedman instigates an altercation while playing a computer game with her 8-year-old brother, Daniel. When Hannah repeatedly disregards her parents’ requests to “please stop screaming” and then also begins to cry, her father, Tom, issues a “time out” command: “Hannah, stop the whining. I want you in your room. Right now.” Hannah’s mother, Alice, reinforces this message in short order by telling Hannah: “You don’t need to scream. You need some quiet time. Go upstairs.” Later, when Hannah is calmer, Tom and Alice remind both children about their expectations for acceptable decorum. “What do we use instead of kicking our hands or feet? What are we supposed to use?” Alice asks the children. “***Words!***” Daniel replies without hesitation. Furthermore, as Tom emphasizes: “Mommy keeps order. That means if people argue and don’t listen, she puts them in time out.” During a CELF research interview, Alice Friedman discusses the importance of maintaining a “sense of balance” with the children. “Because if I’m stressed out,” Alice emphasizes, “they get- they’re stressed.” Further, she avers, “If I’m calmer, ***they’re*** calmer. And they sense it.”

Across town, 4-year-old Jason Goodson’s father initiates a “time out’ for Jason during a Sunday afternoon backyard baseball game. When Jason refuses to relinquish the bat and, in a flash of anger, menacingly swings it toward another child, Chad directs Jason to immediately “put it down, don’t swing it like that!” However, Jason willfully ignores his father’s admonitions and continues to wield the bat. Chad then asks Jason in no uncertain terms: “Do you want to play? Or do you want to go inside?” When Jason responds by angrily hurling the bat across the yard, Chad approaches him, takes him by the hand, and escorts him to his room. “Okay, that’s it,” he apprises Jason. “You don’t throw things at people. You’re not allowed outside for 2 min. You need to stay in your room until then.” When Jason later emerges from his room, Chad inquires of him: “Are you ready? Do you feel better now?” Jason, in response, appearing calmer now, signals his affirmation. Hence, he is permitted to rejoin the game. “If you act the right way,” Chad explains, “then things will happen the right way.” As Chad Goodson later remarks during a CELF research interview, “I think it’s very important to be consistent. To teach him how to calm down.” Jason’s mother, Allison, adds: “It’s important to attain some type of equilibrium that makes everything balanced.”

“Pep talk” and “time out” childrearing practices propel CELF children toward preferred strategies for managing their emotions and, concurrently, toward developing the capacity for self-soothing as a culturally resonant moral technique. These U.S. middle-class childrearing aims exemplify a culturally idealized emotional style that social historian Stearns (1994) terms, “American cool.” Expressions of emotion to trusted figures such as parents are encouraged; however, “emotion must not get out of hand” (Stearns, 1994, p. 191). “One could ‘be oneself,”’ Stearns emphasizes, “only so long as one’s maturity assured that one’s emotions would remain in check and not bother others” (Stearns, 1994, p. 192). These socio-historically shaped American middle-class childrearing practices and ideals, which came to prominence in the mid- to late-twentieth century, emphasize a “restraint of intense emotion” in the interest of “socializing well with others” (Stearns, 2003, p. 73). Significantly, however, as Stearns (1994, p. 4) additionally takes note, although not ubiquitously or uniformly adopted, “emotional culture forms an aspect of middle-class standards that ha(s) some hegemonic power.” These cultural conventions of emotion—or “feeling rules” in sociologist Hochschild (1979, 2012) terms— are resonant with neo-liberal “emotion pedagogies” (Wilce and Fenigsen, 2016, p. 86) that aspire to cultivate “self-managed and self-responsible” persons who learn to regulate their emotions for the benefit of self and others. Middle-class U.S. ethnotheories of emotion, such as those articulated by CELF parents, resonate with professionalized discourses of “positive psychology” (e.g., Seligman, 2011) that champion the merits of emotional temperance and self-regulation in the interest of social connection and “belonging.”

It bears noting that the interpersonal contexts in which “pep talk” and “time out” practices take shape play a central role in conveying the cultural lessons being taught. Emotionally significant relationships hold sway as caregivers situationally and conditionally bestow, or withhold, attention and approval to children in conjunction with their efforts to bolster children’s abilities to suitably manage their own emotions in culturally approved ways. “Pep talk” and “time out” sequences mark overt breaches of conventionalized emotional norms in which children’s visibly (and auditorily) displayed emotional distress is perceived by CELF parents (such as the aforementioned Alice Friedman and Chad and Allison Goodson) to be “out of balance.” These circumstances spark a call to action on parents’ part.

During “pep talk” sequences, parents identify children’s out-of-sorts emotions as attributable to morally accountable reasons that are not of the children’s own making (e.g., as due to others’ oversights or transgressions). In such instances, parents huddle together with children and hold them emotionally and relationally close. In contrast, “time out” sequences are precipitated by children’s untoward emotional (and behavioral) outbursts that are interpreted by parents as signaling a moral breach on the child’s own part. Under these circumstances, parental approbation and attention are conditionally withheld until such time as a child calms down and brings their emotions into check. For example, as CELF mother, Pam Reis, articulates to her 7-year-old son, Mikey, as he oppositionally defies her instructions to finish his homework and raises his fist in her direction: “If you touch me in any way that is not a hug, you’re going to be in bed. That is ***not*** how we express our emotions.” However, in conjunction with both “pep talks’ and “time outs,” children are positioned as responsible moral agents who are charged with affirmatively managing their emotions vis-à-vis their subjectively experienced feeling states and their publicly displayed affective expressions. Moreover, children are accorded moral agency to make socioculturally suitable choices. These core themes are further examined in the extended CELF data sequences that are detailed below.

For example, when 8-year-old Beth Barnes appears restless during her older sister Sonya’s soccer match, her mother, Jacqueline, attempts to calm Beth’s mood by recruiting her into the morally responsible interpersonal task of providing a “pep talk” to 10-year-old Sonya, who is in the midst of a challenging game. “Go tell Sonya she’s doing a good job, sweetie.” Jacqueline further instructs Beth: “Give her some moral support.” In doing so, Jacqueline positions Beth as a morally accountable agent who is capable of productively managing her emotions in the interest of providing encouragement and solace to her older sister, Sonya. Beth is afforded an opportunity to apprentice into the culturally meaningful role of being emotionally attuned and accountable to others.

However, a short while later, Beth’s ill-tempered emotions resurface. “Don’t freak out, sweetie,” Jacqueline advises. However, Jacqueline’s directive to her daughter proves to be of no avail. Beth continues to act disruptively, in a mischievous fashion. When Beth makes several attempts to seize her father Neil’s soft drink out of his hands, he somberly asks Beth: “Are you gonna continue to be ***nasty***, or what?” Neil’s query is met with silence from Beth. When Beth declines to affirmatively choose and endorse the morally preferred course of action, Neil directs Beth to “go stand over there, by that big light pole.” He further advises: “We don’t want you here if you’re going to be acting like that. If you act like that, you can go away. When you feel like you can be civilized, you can come back.” Beth reluctantly backs away in response to her father’s admonishment as she goes to stand next to the appointed light pole. Later, a newly sobered and visibly calmer Beth returns and sidles alongside her father. With lesson learned, she seeks solace in his company once more. The “time out” proposition Neil renders to Beth provides a moral lesson that employs the socializing emotion of disapproval, which is operationalized through Neil’s temporary withdrawal of parental attention to Beth. The moral precept at stake—about how to contain one’s emotions in a “civilized” manner—is thus rendered for Beth in immediate and concrete first-person terms.

Parent-child relations are also at stake in the Morgenstern family. In the following interactional “time out” sequence, 4-year-old Lowell’s mother, Jeri, utilizes the mother-child relationship to motivate a shift in attitude on his part. As the sequence begins, Jeri (line 01) endeavors to help Lowell put on his shoes. Lowell chants raucously as Jeri does so (e.g., lines 02–04, 11–13, 15). When Lowell’s chants grow ever more infelicitous and boisterous (lines 12–13), Jeri leans toward him and catches his gaze (lines 16–17). She then sternly asks Lowell if he needs to “spend some time” in his room (line 18). When he demurs (line 19), Jeri makes it clear to him that she prefers to hear “nice happy words” rather than the type of words he has been using (lines 20–27):

**Table:** 

Data sequence #1		
01	Jeri:	((*seated opposite Lowell, picks up his shoe and loosens the laces*))
02	Lowell:	Put my ***shoes*** on, put my stinky ***shoes*** on! ((*loudly chants*))
03		Put my stinky ***shoes*** on! Ah, ah, ah. Stinky ***shoes***, ((*chants*))
04		Stinky ***shoes***. Put my stinky ***shoes*** on! ((*laughs, kicks feet*))
05	Jeri:	Is that a new song? ((*gazes intently at Lowell*))
06		I never heard that song before. ((*begins to put on Lowell’s shoe*))
07	Lowell:	Mhm. Hhh. ((*laughs*))
08		It’s too tight! I think ***I*** want to put my shoes on.
09		((*takes shoe from Jeri*))
10		***I*** put it. ((*attempts to further loosen shoelace*))
11		Big fat liar. ((*chants while attempting to loosen shoelace*))
12		Big fat LIAR! ((*chants in a louder voice*))
13		[Big fat LIAR! Big fat ***LIAR!*** ((*chants boisterously*))
14	Jeri:	[Hey, hey, *hey!* ((*turns toward Lowell, speaks sternly*))
15	Lowell:	You’re a big fat ***LIAR = !***
16	Jeri:	= Lowell! Lowell! ((*leans toward Lowell, gazes directly at him*))
17	Lowell:	What = ? ((*returns Jeri’s gaze*))
18	Jeri:	Do you need to spend some time in your room? ((*stern voice tone*))
19	Lowell:	No. ((*averts gaze*))
20	Jeri:	Cause the words that are coming out of your mouth
21		Are ***not*** nice happy words. ((*gazes at Lowell, leans in closely*))
22		[Okay = ? ((*re-establishes eye contact with Lowell*))
23	Lowell:	[((*fidgets in his seat, giggles and slyly smiles*))
24	Jeri:	You have a big smile on your face.
25		((*gazes directly at Lowell*))
26		I want to hear nice ***happy*** words.
27		I don’t want to (.) hear ***those*** words. Okay = ? ((*gazes at Lowell*)
28	Lowell:	= The Big Fat Liar is a movie. Hhh. ((*meets Jeri’s gaze, laughs*))
29	Jeri:	It actually is. Hhh. ((*laughs*))
30		((*Lowell and Jeri continue their discussion about the movie…*))

When Lowell’s boisterous chants about his “stinky ***shoes***” (lines 02–04) become ever more discourteous (e.g., lines 13, 15: “Big fat LIAR! Big fat ***LIAR!*** You’re a big fat ***LIAR!***”), in Jeri’s view, she leans in toward Lowell and repeatedly catches and holds his gaze in a direct facing formation (e.g., lines 16–17, 21, 25). Jeri’s embodied countenance and her stern tone of voice (e.g., lines 14, 16, 18) communicate to Lowell her seriousness of purpose. When Jeri, in line 18, asks Lowell: “Do you need to spend some time in your room?” she positions him as a morally accountable actor who is expected to manage the emotions he expresses, along with the behaviors that flow from these emotions, in socially responsible ways. Jeri next reflects back to Lowell the discrepancy she perceives between his facial expression and the feeling tone of the words he is speaking (lines 20–22, 24–27). She also explicitly articulates her expectations by telling Lowell in no uncertain terms (lines 26–27) that, “I want to hear nice ***happy*** words. I don’t want to (.) hear ***those*** words.” Additionally, Jeri seeks affirmative uptake from Lowell (line 27: “Okay?”). In doing so, she again positions Lowell as a morally accountable agent who is called upon to assume responsibility for his response. The relational fulcrum—in which parental approval and attention are potentially at stake—provides an interactionally salient means of leverage that helps prompt the emotional tenor of the action trajectory toward a more lighthearted tone. Lowell’s unhesitating convivial response in line 28 (“=The Big Fat Liar is a movie.”), which he accompanies with laugher, improvisationally shifts the interaction’s referential focus by reframing the meaning and purpose of the words that Jeri (lines 20–21) has previously delineated as “***not*** nice happy words.” The contested words “big fat liar” consequently are recontextualized and are thus provided with a culturally acceptable warrant and purpose by virtue of their association with a popular movie that Lowell and Jeri can amicably discuss with one another (lines 28–30).

Several days later, when Jeri’s 8-year-old daughter Anna becomes emotionally distraught while experiencing difficulties with her homework, Jeri again employs a “time out” strategy. In doing so, Jeri prompts Anna to get a handle on her emotions. When Jeri’s initial efforts to help calm her daughter prove to be of no avail (lines 01–31, below), she proposes a “time out” by baldly asking Anna: “Do you need to go to your room?” (line 32). When Anna continues to behave defiantly (e.g., lines 33, 39). Jeri informs her that, “this is not appropriate right now” (lines 35–38). She then lays out a series of definitive choices for Anna, which communicate her expectation that Anna respond as a morally accountable agent (lines 40–41): “Are you going to finish your homework? Or go to your room? Those are your choices. It’s your decision.” When Anna once more equivocates (line 42), Jeri again proposes a “time out” for Anna (line 43: “Do you want to take a few minutes?”). This sparks a reevaluation on Anna’s part and facilitates Anna’s capacity to regain her composure and subsequently resume her homework (lines 44–46):

**Table:** 

Data sequence #2		
01	Anna:	What’s the answer? I ***don’t*** know it. I don’t know what that is.
02		Hhh hh h. ((*begins to cry*))
03	Jeri:	Let’s try together. ((*calm voice*))
04		It’s in between the twelve and the one, so it’s-
05	Anna:	One?
06	Jeri:	It’s not ***on*** the one. It’s in ***between*** the twelve and the one.
07		So it’s going to be?
08	Anna:	I don’t ***know***. This is hard work. ((*exasperated voice tone*))
09	Jeri:	You need to hang in there.
10		You’re not done yet and you’re starting to lose focus.
11		Do you want something to drink?
12	Anna:	No. I want something to eat.
13	Jeri:	Well, dinner is going to be ready soon.
13		And I don’t want you to spoil your appetite.
15	Anna:	((*walks to kitchen cabinet, reaches for cereal box*))
16	Jeri:	No honey, I don’t want you having cereal.
17		We’re going to eat in a few minutes.
18	Anna:	I need ***something***. Hh hh hh. ((*cries*))
19	Jeri:	Anna, Anna, listen to me = . ((*firm voice tone*))
20	Anna:	= Please, please, please! ((*pleads, approaches Jeri and hugs her*))
21	Jeri:	How about some lemonade = ? ((*hugs Anna*))
22	Anna:	= ***No!*** ((*defiant voice tone*))
23	Jeri:	It will fill you up until-
24		How about some grapes?
25	Anna:	No. ((*walks to kitchen cabinet, takes out cereal box*))
26	Jeri:	You’re not having cereal right now before dinnertime.
27		It’s going to fill you up.
28	Anna:	No it ***isn’t!*** ((*pouts*))
29		Please, please, please! Hhh hh. ((*begins to cry*))
30	Jeri:	You can have an apple. Or some grapes? ((*offers fruit to Anna*))
31	Anna:	***No***. I don’t ***want*** that. ((*pushes fruit away*))
32	Jeri:	Do you need to go to your room?
33	Anna:	No. I need a ***pretzel***. ((*defiantly*))
34	Jeri:	You’re going to end up in your room.
35		And you’re going to end up not coming out all night, okay?
36		Because this is not appropriate right now and I don’t know what
37		the drama is about but I can guess since I know you’re tired and
38		hungry. You have to wait a few minutes and be patient.
39	Anna:	I want something to ***eat***. ((*insistent voice tone*))
40	Jeri:	Are you going to finish your homework? Or go to your room?
41		Those are your choices. It’s your decision. ((*firm voice tone*))
42	Anna:	I don’t know. I’m just ***starving***. ((*distraught*))
43	Jeri:	Do you want to take a few minutes?
44	Anna:	No, I don’t. ((*quiet voice tone*))
45	Jeri:	Okay. Then come finish your homework.
46	Anna:	Okay. ((*takes a seat at kitchen table, calmly resumes homework*))

In responding to Anna’s palpable emotional distress, Jeri makes efforts to establish and display empathic attunement with Anna so as to help relieve her daughter’s expressed disquietude (e.g., lines 02, 09–11, 21–24, 30, 37–38). However, Anna continues to appear inconsolable and emotionally distraught (e.g., lines 01, 08, 12, 15, 18, 20, 22, 25, 28–29, 31). Jeri’s suggestions of a “time out” for Anna are intermittently couched as questions, in indirect terms (lines 32, 35, 40). These are posed in conjunction with Jeri’s baldy direct parental edicts (e.g., lines 34, 36, 41) and as such soften their authoritative tone. This discursive combination of parental authority and emotional support provides a structured yet empathically engaging interactional exchange in which Anna is encouraged to responsibly manage her emotions in socially permissible ways. As the sequence closes, Anna, now visibly calmer, re-attains an alignment of perspectives with her mother and also successfully carries on with her homework (lines 43–45).

In another CELF household across town, 8-year-old Tara Lear fusses when her mother, Cheryl, encourages her to share a new toy with her younger sister Cassie (age six). “Why does ***she*** have to see it?” Tara indignantly exclaims. Cheryl proposes an alternate solution: “Come sit on the floor and look at it together. It’s more fun doing things together.” “I don’t ***care!***” Tara exclaims. She hurls the toy to the floor and menacingly sticks out her tongue. When her sister Cassie retrieves the discarded toy, Tara bats it from Cassie’s hands and reclaims it for herself. “Tara! One more time and you’ll be in your room,” her mother warns. Notably, as in many of the “time out” data sequences evident in the CELF data corpus, Cheryl poses a conditional set of options from which Tara is expected to choose. Tara and other CELF children (such as Lowell and Anna Morgenstern) are thus construed as morally responsible actors who are encouraged to handle their emotions, along with their ensuing actions, in culturally endorsed ways. Moreover, parents take pains to actively guide children toward culturally preferred moral choices in which “smooth relations with others” (Stearns, 1994, p. 190) play a paramount role.

During a CELF research interview, Tara and Cassie’s parents talk about how they encourage the girls to take active charge of their emotional lives by “chang(ing) their mood” to “help create better balance.” The girls’ father, Adam, emphasizes that, “it’s important to instill a positive attitude so the kids grow up feeling confident about themselves and good about themselves.” Cheryl also notes that, “it’s important to not keep things bottled up inside. It’s important to talk it out. … If they keep it inside, I just think that would be damaging.”

Cheryl translates this ideology into action several days later when she invites 10-year-old Cassie to “talk out” her feelings about an upsetting incident that transpired with a school classmate. When Cheryl discovers Cassie crying in her room, she prompts Cassie to articulate her feelings and identify the source of her distress (lines 01–04, below). When Cassie endorses potent feelings of anger as well as sadness (line 05), Cheryl engages Cassie in a problem solving pep talk, as follows in the data sequence below:

**Table:** 

Data sequence #3		
01	Cassie:	Hhh hhh hh. ((*sobs, with face buried in hands*))
02	Cheryl:	Why are you crying? ((*soft, soothing voice tone*))
03		(1.0)
04	Cheryl:	Why are you crying? ((*reaches out, gently caresses Cassie*))
05	Cassie:	I ***hate*** Amelia.
06	Cheryl:	What? Can you tell me the whole story?
07	Cassie:	On Monday when I wore that skirt to school, Amelia walks up and
08		says, “you ***copied*** my skirt.” And I’m all, “no, I didn’t!” And she’s
09		all, “yes you ***did!***” And she’s all, “nobody could have the exact
10		same skirt as me. ***I*** don’t like it!” I feel like she said to me “you
11		should go ***return*** the skirt.” I thought Amelia was ***nice***. But she’s
12		acting more ***rude*** than nice. To especially ***me***.
13	Cheryl:	Don’t let Amelia tell you what you ***can*** and ***cannot*** wear and make
14		you feel bad. And I know it does. You can tell her “you don’t have
15		to ***let*** me wear the skirt. I can wear ***any*** skirt I like!” And you don’t
16		have to be Amelia’s friend. You don’t have to be ***mean*** to her but
17		you ***don’t*** have to be her friend. ((*gently strokes Cassie’s back*))
19	Cassie:	That’s why I got really ***mad*** inside. Hhhh hh hhh. ((*sobs*))
20	Cheryl:	Hmm. It’s ***okay***. ((*continues stroking Cassie’s back*))
21	Cassie:	[I just got really mad that I had to write it.
22		[((*displays crumpled paper on which she has written*))
23	Cheryl:	That’s- that’s a ***great*** way to get your feelings out as long as
24		somebody doesn’t see it. … You can ***write*** it and rip it ***up***. And
25		write it and ***show*** it to me. Or ***talk*** to me about it. Okay?
26	Cassie:	((*nods*))
27	Cheryl:	It’s ***better*** not to keep it inside. …
28		You can ***always*** tell me if something like that happens, okay?
29	Cassie:	((*nods head*))
30		(0.5)
31	Cheryl:	Do you feel better talking about it?
32		((*sustains eye gaze with Cassie*))
33	Cassie:	((*nods head, returns Cheryl’s gaze*))
34	Cheryl:	And don’t you- (.) you see that Amelia’s being ***not*** nice.
35		It’s not ***you***, it’s ***her***. …
36	Cassie:	But it’s ***mean***=.
37	Cheryl:	= ***Very*** mean.
38	Cassie:	Hhhh hhh hhhh. ((*resumes crying*))
39	Cheryl:	Come here. ((*soothing voice tone, hugs Cassie*))
40		I ***love*** you. I’m so ***proud*** of you. ((*continues hugging Cassie*))
41	Cassie:	((*hugs Cheryl, in return*))
42	Cheryl:	***Amelia’s*** got a problem, not you. Okay?
43	Cassie:	Okay. Hhh. ((*quiet voice tone, takes a deep breath*))
44	Cheryl:	It’s not your problem. I hope you ***know*** that. ((*gazes at Cassie*))
45	Cassie:	((*returns Mother’s gaze, nods head*))

During this heart-to-heart mother-daughter talk, Cheryl encourages Cassie to articulate her feelings, directly and unabashedly (e.g., lines 02, 04, 06, 23–25, 27–28). Cassie’s mother guides her in translating her feelings into self-protective assertive action rather than using them to wound others (e.g., lines 13–17, 23–25). Moreover, she takes pains to deter Cassie from internalizing a sense of self-blame, lest Cassie regard *herself* as “a problem” (lines 27–28, 34–35, 44). Cheryl thus articulates her own sense of pride in her daughter, which is delivered in tandem with a morale boosting hug and a motherly expression of love (e.g., lines 39–41). Cheryl’s corporeal engagement with Cassie throughout the exchange—via comforting embraces, soothing voice tones, and direct eye-to-eye contact—facilitate Cassie’s ability to contain the strong feelings she is experiencing and to regain a sense of efficacy and pride (e.g., lines 33, 43, 45).

CELF parents Jacqueline and Neil Barnes espouse a parenting philosophy that likewise incorporates morale bolstering strategies on their daughters’ behalf. Says Jacqueline: “I want them to have confidence in themselves.” This childrearing approach comes to the fore, for example, during 8-year-old Beth’s Saturday morning soccer game. When Beth expresses disappointment over her performance as the team’s goalie, Jacqueline joins her on the field and engages Beth in a confidence strengthening “pep talk.” In the data sequence that follows, Beth (line 01, below) appears dejected when Jacqueline arrives at her side. Jacqueline, in turn, offers Beth reassurance and consolation, which she expresses through her words as well as through her embodied demeanor (e.g., lines 02–03, 05–06, 13):

**Table:** 

Data sequence #4		
01	Beth:	[((*approaches mother, appears distraught*))
02	Jacqueline:	[Hey sweetie, you did such a good job! ((*approaches Beth*))
03		I’m so ***proud*** of you! ((*cheerful voice tone*))
04	Beth:	But they scored a ***goal***. ((*distressed voice tone*))
05	Jacqueline:	There was no ***way*** you could have gotten that.
06		That was so ***high***. There was no way. ((*leans down, kisses Beth*))
07	Beth:	I ***touched*** it =.
08	Jacqueline:	Yeah, but it was just too ***high*** for you.
09		((*enfolds Beth in her arms, picks her up and carries her*)) *…*
10		It’s stressful being goalie, right?
11	Beth:	Yeah. ‘Cause if they score- = ((*looks downward*))
12	Jacqueline:	You feel like it’s your fault.
13		But, it’s really ***everybody’s*** fault, right? ((*gazes toward Beth*))
14	Beth:	((*returns Jacqueline’s gaze*))
15	Jacqueline:	That was a good game against the ***hardest*** team.
16		They played- they played their hearts out.
17		So ***good*** game!
18	Beth:	((*nods, sheepishly smiles*))

When Beth (line 01) appears downcast following her soccer game, Jacqueline provides solace. “Hey sweetie, you did such a good job! I’m so ***proud*** of you!” she enthusiastically exclaims (lines 02–03). Beth (line 04), however, counters her mother’s assessment of the situation: “But they scored a ***goal***,” she protests. In response, Jacqueline proffers a face-saving rationale (line 05): “There was no ***way*** you could have gotten that,” she emphatically tells Beth. “That was so ***high***. There was no way,” Jacqueline adds (line 06). She kisses Beth and scoops Beth into her arms. “It was just ***too*** high for you,” Jacqueline remarks, as she carries Beth across the field (lines 08–09). “It’s stressful being goalie, right?” Mom (line 10) later inquires of Beth. “Yeah,” Beth concurs (line 11). “‘Cause, if they score-” Beth’s voice trails off, mid-sentence, and Jacqueline steps in to finish the thought (lines 11–12): “You feel like it’s your fault.” “But,” she continues on (lines 13, 15–17), “it’s really ***everybody’s*** fault, right? That was a good game against the ***hardest*** team. … So ***good*** game!” The pep talk Jacqueline provides for Beth is designed to sooth her disappointed feelings and to bolster her sense of efficacy. During the interactional sequence, Beth listens intently (e.g., line 14) and she responds to her mother with a nod and a smile (line 18). However, as Beth reflexively reevaluates the situation and her feelings about it during a subsequent CELF research interview, she articulates a more complex, emotionally ambivalent point of view. For instance, as Beth remarks later during the research interview:

It’s scary being a soccer goalie. And, last game I didn’t stop one ball. My mommy says I’m great but I don’t know if I’m good or not. … I believe my mom but I still think she may be saying that to make me feel better.

In other instances, CELF research data chronicled children’s wholehearted uptake of their parents’ “pep talk” endeavors, which were intended to boost children’s motivation, determination, and self-esteem. For example, 10-year-old Leslie Walters provides a motivating pep talk on her own behalf that is geared toward revitalizing her lagging school performance. Leslie speaks aloud within the earshot of other family members, as follows:

I’m going to show him (Leslie’s athletic coach), and I’m going to say: “I’M NOT BAD IN GRADES!” I’m going to just really improve ‘cause I ***really*** want to get a good grade. And I’m going to be, “wow!” I’m going to try to do my best, take my time, and do my best!

Several days later, upon receiving news of Leslie’s gradually improving academic performance, Leslie’s mother, Lila, provides her with further encouragement by initiating a mother-daughter “pep talk.” Lila lauds Leslie as she enthusiastically observes, “Honey, this is the ***best*** report card you’ve ***ever*** had!” Leslie responds by musing to her mother: “I think that’s the hardest I ever worked!” “And, look at the ***results***!” Lila zealously exclaims. Leslie smiles broadly as she contemplates her mother’s heartening words. “But you see, honey,” Lila underscores, “the hard work isn’t for ***nothing***, is it?” Leslie concurs by nodding her head emphatically. “I just need to try extra, extra hard!” she concludes. Leslie’s self-motivating words are accompanied by a noticeably brightened mood and a tone of determination and self-assurance.

The moral lessons that inhere in the parent-child “pep talk” and “time out” practices explored in the preceding CELF data sequences hold sway to influence children’s culturally shaped *oeuvres* of emotional resources and responses. As demonstrated above, such practices shape the evaluative frameworks through which children learn to read and respond to others’ emotions. In line with these data findings, I propose that interpersonal relations, affect, and morality are critical intertwined dimensions of cultural acquisition as children navigate landscapes of emotion in tandem with those in their social surround. The intimate interpersonal relationship between parent and child serves as a resource to scaffold and encourage children’s attentional focus on the culturally and morally preferred affective dispositions, orientations, and stances that are being mentored. The parent-relationship thus provides a motivational impetus that propels children to further action and mastery of the emotionally and morally resonant lessons at hand. Parental disapproval—and the temporary prohibition of children from the close physical proximity and positive emotional attention of parents (and others)—are used as negative reinforcements in conjunction with “time outs” in motivating children to refrain from unwanted behaviors and to learn salient lessons about culturally preferred affects, attitudes, and behaviors. Emotion within the context of the parent-child relationship is thus used to mark important moral lessons in affectively noticeable and memorable ways.

By closely considering culturally entwined aspects of morality and emotion, we are spurred to contemplate how emotions and their moral dimensions may operate as lodestones that draw people together or, alternatively, may serve as barricades that separate people from one another. Additional theoretical and practical implications that flow from these research findings, along with potential directions for additional future research, are explored in the concluding section below.

## Concluding Remarks

The research findings explored above sketch out the contours of two culturally shaped discursive framings of emotion regulation employed by CELF families to mentor children into culturally preferred value orientations in the contexts of their daily lives. In this section, I further consider how these discursive configurations of emotion and morality, which CELF family members term “pep talks” and “time outs,” serve as occasions for cultural learning that involve the transmission and shaping of culturally desirable aspects of moral personhood and relationality. As the preceding data examples illustrate, emotions employed within the context of the parent-child relationship during “pep talk” and “time out” sequences communicate and model to children culturally pertinent moral norms regarding the expression and management of emotions. It is argued that the emotional salience of the parent-child relationship supplies a motivational impetus that potentiates these opportunities for apprenticing children into salient practices, techniques, and norms of emotional regulation and expression.

The current research suggests that parental communications to children that convey praise and approval, or that conversely relay disapproval, are emotionally resonant motivational practices in this U.S. middle-class cultural milieu. Children are thus urged to heed caregivers’ emotionally salient, interpersonal appeals. As they do so, they are mentored into morally resonant techniques for managing their emotions, such as self-soothing practices that make use of positive uplifting self-talk. Significantly, as well, when caregiving adults recognize, appraise, and respond to children’s outward signs of emotional distress or perturbation, caregivers are themselves engaged in a mutually shaped moral project that engages “institutionally and culturally informed techniques” for rectifying children’s “troubles” and for “guiding their behavior” (Kidwell, 2011, p. 262). As they do so, caregivers employ a broad variety of sensory modalities, including touch, talk, gaze, and other proprioceptive capacities to facilitate a “recalibration of the child’s emotional state” (Cekaite and Kvist, 2017, p. 127). This involves cultural learning that transpires at the “level(s) of form, movement, feeling, and sentiment” (Schwartz, 1976, p. xi).

Notably, however, “time outs,” in particular, were rarely employed with CELF toddlers (age two and younger). CELF parents instead were inclined to offer such youngsters affective and corporeal comfort and reassurance (such as holding, cuddling, and/or distracting) to calm their ruffled emotions. This data finding suggests that CELF parents held different developmental expectations for these younger children as compared with older children. Although the number of these younger children among the CELF data sample is relatively small (*n* = 5), these findings are suggestive of a promising direction for additional future research. When “time outs” were employed for socialization purposes with pre-schoolers and kindergarteners (ages three to five), CELF parents commonly employed a direct (e.g., gaze-to-gaze) facing formation to help secure and maintain their child’s attention, in combination with concrete tactile and corporeal cues (e.g., such as physically guiding children), as did 4-year-old Jason Goodson’s father in the data example detailed earlier. Although “pep talks” were used across all age groups, they were most commonly employed with CELF school-aged children and young adolescents (ages five to thirteen). “Time outs,” likewise, were most often used with this same age group who presumably possess more fully developed linguistic capacities and cognitive reasoning skills in comparison with younger children. “Pep talks” and “time outs” were employed less frequently with CELF older adolescents (ages fourteen to sixteen). This finding implies that CELF parents may expect these youth to have more fully internalized the culturally and morally prescribed expectations and skills of emotion management.

The CELF “pep talk” and “time out” data examples suggest that motivationally salient discourses of emotion resonate with, and reinforce, consensually shaped “cultural models of virtue” (LeVine and Norman, 2001, p. 84). Such moral orientations, Shweder (2012, p. 91) argues, are “motivators of action in significant measure because they are affect-laden and produce in people powerful feelings of arousal, distress, pollution, repugnance, guilt, indignation, pride, or shame.” These are enacted across a range of CELF family activities and occasions, so as to mentor children into culturally valued stances, actions, competencies, and worldviews. The psychoculturally relevant themes that come to the fore in conjunction with “pep talk’ and “time out” practices apprentice children into morally accountable ways of managing their emotions vis-à-vis their relations with others. These practices unfold amidst the culturally rooted “interpersonal space of related selves” that U.S. middle-class family life provides (Jenkins, 1991, p. 389). The socio-emotional climate that is fostered in conjunction with these emotionally pertinent moral “dramas” (Briggs, 1998) encompasses a suite of thematically and affectively linked value orientations, dispositions, and stances that are embodied and expressed across various domains of family life as children and parents take up—and reciprocally shape— culturally available resources, priorities, and scripts (cf. Goodwin et al., 2012).

The current analysis highlights culturally viable strategies employed by contemporary U.S. middle-class families for propelling children along cultural pathways for success. I argue that these “pep talk” and “time out” childrearing practices accord with what sociologist Lareau (2011) has termed, “concerted cultivation,” whereby children’s capacities and skills are purposively “cultivated” through active intervention and guidance. The “pep talk” and “time out” practices evident in the CELF data corpus fit with Lareau’s conceptual characterization of “concerted cultivation” in that they are consciously and strategically used by CELF parents to develop and shape children’s *oeurvre* of culturally and morally approved affective resources and skills. “Pep talk” and “time out” practices also have resonance with what anthropologist Kusserow (2004, 2005)—in her study of U.S. childrearing and social class—describes as “soft individualism.” Kusserow found that this childrearing strategy was prevalent among the New York middle-class families she studied. “Soft individualism,” according to Kusserow (2005, p. 40) focuses on cultivating children’s “unique feeling, thoughts, ideas, and preferences.” Likewise, the “pep talk” and “time out” childrearing techniques used by CELF parents are geared toward developing children’s discursively constructed selves, subjectivities, self-awareness, and points of view.

In closely tracking CELF research participants’ discursive framings of normativity and moral accountability, it is important to attend to how each discursive frame incorporates—and shapes—deeply held sentiments and institutionally sanctioned values (cf. D’Andrade, 2008) vis-à-vis preferred cultural pathways for facilitating children’s ongoing success and wellbeing. I argue that both discursive strategies (“pep talks” and “time outs”) embody culturally warranted aims for motivating U.S. middle-class children along a pathway toward success in a post-industrial societal context that champions moral accountability toward others in addition to oneself. Such on-the-ground practices are compatible with broader macro-level discourses and master narratives, such as those associated with a neo-liberal emphasis on cultivating citizens who learn to regulate their emotions on behalf of self and others (cf. Rose, 1998; Wilce and Fenigsen, 2016). In a related vein, sociologist Friedman (2013, p. 3) observes that U.S. middle-class families have developed a robust set of resources and strategies intended to facilitate children’s abilities to cultivate and maintain amicable, stable interpersonal relationships amid a broader social landscape in which U.S. middle-class children’s socioeconomic futures are far from secure. Friedman (2013, p. 3) speculates that this trend stems from “middle class insecurity and concerns about children falling behind”.

Importantly, however, formal and informal parental ethnotheories (Fung, 1999) of childrearing—and the cultural contexts in which they are employed, reinforced, adapted, and transformed—are most often “complex and multifaceted,” rather than unambiguously uniform; as such, they facilitate developing children’s capacities to flexibly, creatively, and adaptively “function in a dynamic and fluid society (Edwards et al., 2006, p. 149). In this respect, “pep talk” and “time out” practices are most accurately conceptualized as two points on an interrelated continuum rather than as mutually exclusive, discrete cultural alignments.

Cultural schemas of normativity—and their culturally resonant emotional, moral, and interpersonal orientations, qualities, dispositions, and worldviews—are aimed toward enhancing children’s wellbeing and optimal development in relationship with the cultural worlds in which children reside. Cultural schemas, which involve experientially mediated “clusters of strong associations,” are prototypically catalyzed in conjunction with heightened emotions such as those that are sparked through emotionally galvanizing childrearing practices (Quinn and Mathews, 2016, p. 359). It is proposed that this process takes place during CELF “pep talk” and “time out” data sequences in which morally charged affective stances play a role in shaping culturally preferred patterns of feeling, meaning, and behaving. Through their recurrent participation in “pep talk” and “time out” practices, CELF children learn to attend to particular embodied feeling states and to associate these feelings with culturally shaped configurations in which co-occurring sentiments, actions, and interpersonal inclinations are regularly paired with one another. Such practices apprentice children into moral accountable relationships with others by encouraging them to manage their emotions in culturally and socially preferred ways. The children are thus mentored into culturally consonant moral techniques that involve identifying and responding to the emotions of self and others, such as practices that incorporate and encourage children’s emotional self-soothing and motivational self-talk.

Affectively laden “pep talk” and “time out” childrearing practices incorporate key factors that psychological anthropologist Quinn (2005) specifies as facilitating children’s successful developmental transformation into culturally valued adults. Quinn (2005, p. 477) posits that emotionally memorable, re-occurring, and thematically coherent cultural lessons such as these—which involve “emotional arousal” and caregivers’ “evaluations of the child as approved or disapproved of”—enhance children’s overall receptivity to the lessons-at-hand by contributing to the lessons’ unmistakability, durability, and motivational salience.

These culturally shaped moral priorities are explicitly and tacitly communicated, embodied, reinforced, and adapted as children and their parents co-participate in recurrent family activities and routines. Importantly, however, as is evident in the CELF data sequences, children and parents actively and improvisationally operate upon these cultural messages by imbuing them with shades of personal significance and meaning (also see Goodwin et al., 2012). Such “day-to-day workings of family life” (Ochs and Kremer-Sadlik, 2013b, p. 237) provide crucial clues as to how moral sentiments, stances, outlooks, and preferences come into fruition and are transacted in everyday lived contexts of use. Moreover, as is demonstrated by the current study’s findings, this on-the-ground view of morality as it is lived and negotiated within naturalistic, interpersonal contexts serves to augment traditional understandings of morality and ethics as derived from more circumscribed methodological approaches.

Rogoff et al. (2018, p. 5) emphasize that the ecological validity of research findings about child development necessitates the direct study of children’s naturalistic participation in day-to-day practices and settings, and amidst the cultural communities in which their everyday lives are lived. The current study’s naturalistic ethnographic data findings are interesting to consider in tandem with the psychological research findings of Wang and Fivush (2005); also see Wang (2006) that employ elicited narrative data to explore parents’ strategies for mentoring children into culturally salient emotion regulation techniques during parent-child conversations about emotionally noteworthy positive and stressful events. Wang and Fivush found that Euro-American mothers employed a “cognitive approach” that emphasized the use of explanatory rationales to assist children’s sense-making and emotion regulation capacities whereas Chinese mothers employed a “behavioral approach” that emphasized proper conduct and that assisted children to regulate emotions by building their affiliations with others and by recognizing and adjusting to social norms. CELF parents’ incorporation of “pep talks” and “time outs” into their children’s day-to-day lives as culturally informed techniques for mentoring children into morally preferred practices of emotion regulation employ a combination of discursive and non-discursive *genres* that combine narrative self-reflection with socially embedded moral action, rather than employing mutually exclusive approaches.

However, the CELF study’s modest number of research participants constitutes an important caveat that predisposes against any further, widespread generalization of the key research findings presented here. The current data findings are considered to be preliminary. For example, additional cross-cultural comparison is a productive avenue for further, ongoing research. Discernable differences in gendered participation in “pep talk” and “time out” practices were not evident in the data, either among CELF children or among CELF parents. However, this is another fruitful area for continued future research in light of the CELF study’s limited sample size. Research participants’ potential video reactivity is another of the study’s potential limitations. Albeit pertinent research data are generated even when participants are modeling “ideal typical” behaviors and affects, in that these are representative of what research participants take to be indicative of normative behaviors, affect, and utterances in accordance with local cultural schemas of such. The current study’s findings are to be interpreted in light of a heterogeneous conception of culture, which construes it as a set of lived processes that conjoin individual lives with those of others in mutually recognizable ways but that also affords possibilities for individual, social, and historical variation and change.

## Ethics Statement

This research was conducted in accordance with the recommendations of the Institutional Review Board at the University of California, Los Angeles (UCLA). The research protocol was reviewed and approved by the UCLA Institutional Review Board (UCLA IRB Protocol #G01–06–083–14). Written informed consent (or assent) was provided by research participants in accordance with the World Medical Association’s Declaration of Helsinki. Study participants’ names have been altered to safeguard confidentiality as per IRB-approved research protocol.

## Author Contributions

KS completed the fully detailed transcription of the discourse data that are analyzed and discussed in this article so as to facilitate the identification of relevant discourse phenomena and to enhance the granularity of the analysis. Additional rounds of data analysis and coding were conducted by KS using a “grounded theory” approach (Corbin and Strauss, 2008; Glaser and Strauss, 2017) to identify pertinent interactional patterns and features that organically and empirically emerged from the data. All data analysis procedures were conducted by KS. KS solely conceptualized this research topic and focus of analysis.

## Conflict of Interest Statement

The author declares that the research was conducted in the absence of any commercial or financial relationships that could be construed as a potential conflict of interest.

## Appendix: Transcription Conventions

The following transcription conventions, adapted from Atkinson and Heritage (1984), are employed in this article to demarcate conversational phenomena. A more fully detailed transcription of the discourse data (based upon Atkinson and Heritage, 1984) was also completed by KS to facilitate accurate identification of relevant discourse phenomena and to enhance the granularity of the data analysis.

**Table:**

***word***	Bold italics indicate emphasis, such as changes in pitch and/or amplitude.
WORD	Capital letters indicate increased volume.
-	A hyphen after a word or part of a word indicates a cut-off or interruption.
=	An equals sign signifies utterances that occur in quick succession.
[	A left bracket indicates the point at which speakers’ utterances overlap.
(1.5)	Numbers enclosed in parentheses represent silences, measured in seconds.
(.)	A dot in parentheses denotes a micropause, two-tenths of a second or less.
Hhh	“Hhh” signifies audible aspiration (such as laughter or crying).
…	Ellipses demarcate elisions of circumscribed portions of the dialogue.
(( ))	Double parentheses indicate descriptions and/or commentary on the data.
